# Proteomic Analysis of Hypothalamus and Pituitary Gland in Pre and Postpubertal Brahman Heifers

**DOI:** 10.3389/fgene.2022.935433

**Published:** 2022-06-14

**Authors:** Loan To Nguyen, Li Yieng Lau, Marina Rufino Salinas Fortes

**Affiliations:** ^1^ Queensland Alliance for Agriculture and Food Innovation, The University of Queensland, Brisbane, Australia; ^2^ Agency of Science, Technology and Research, Singapore, Singapore; ^3^ School of Chemistry and Molecular Biosciences, The University of Queensland, Brisbane, Australia

**Keywords:** puberty, hypothalamus, pituitary gland, brahman heifers, proteomics

## Abstract

The hypothalamus and the pituitary gland are directly involved in the complex systemic changes that drive the onset of puberty in cattle. Here, we applied integrated bioinformatics to elucidate the critical proteins underlying puberty and uncover potential molecular mechanisms from the hypothalamus and pituitary gland of prepubertal (*n* = 6) and postpubertal (*n* = 6) cattle. Proteomic analysis in the hypothalamus and pituitary gland revealed 275 and 186 differentially abundant (DA) proteins, respectively (adjusted *p*-value < 0.01). The proteome profiles found herein were integrated with previously acquired transcriptome profiles. These transcriptomic studies used the same tissues harvested from the same heifers at pre- and post-puberty. This comparison detected a small number of matched transcripts and protein changes at puberty in each tissue, suggesting the need for multiple omics analyses for interpreting complex biological systems. In the hypothalamus, upregulated DA proteins at post-puberty were enriched in pathways related to puberty, including *GnRH, calcium and oxytocin signalling pathways*, whereas downregulated proteins were observed in the *estrogen signalling pathway, axon guidance and GABAergic synapse*. Additionally, this study revealed that ribosomal pathway proteins in the pituitary were involved in the pubertal development of mammals. The reported molecules and derived protein-protein networks are a starting point for future experimental approaches that might dissect with more detail the role of each molecule to provide new insights into the mechanisms of puberty onset in cattle.

## Introduction

Early puberty is essential for the lifetime reproductive performance of cattle (Lesmeister et al., 1973; Johnston et al., 2013). However, Brahman cattle, a breed of the *Bos indicus* sub-species, which can withstand hostile conditions in northern production systems, are often older and heavier at puberty than *Bos taurus* breeds (Chenoweth 1994). As reaching the age of puberty is an important event contributing significantly to lifetime productivity, reducing the age at puberty is a major aim for *Bos indicus* breeders for efficient herd productivity.

Like other vertebrates and humans, puberty in cattle is initiated when the hypothalamus-pituitary-ovary (HPO) axis loses its sensitivity to negative feedback effects of steroid hormones, allowing an increase in gonadotropin-releasing hormone (GnRH) secretion from GnRH neurons in the hypothalamus. The increase then stimulates gonadotropins’ secretion: luteinizing hormone (LH), and follicle-stimulating hormone (FSH) are produced and released. FSH and LH then regulate gonadal development (Day et al., 1984; Day et al., 1987; Schillo et al., 1992; Day and Anderson, 1998; Gasser et al., 2006). However, the process is controlled by multiple factors, and the complex interactions between environmental and genetic factors regulating the process are only now coming to light.

The complex genetic architecture of puberty–that is multiple variants of small effect–is probably underpinned by variants with transcriptional and post-transcriptional regulatory effects. Variations in the expression of genes or proteins in the hypothalamus or the pituitary gland, either alone or simultaneously, will affect the pubertal process. To date, several transcriptomic studies have focused on investigating candidate molecules involved in critical molecular mechanisms that drive puberty in *Bos indicus* in the HPO axis. Fortes et al., 2016 identified five transcription factors, *E2F8, NFAT5, SIX5, ZBTB38,* and *ZNF605,* with potential regulatory roles at puberty in the hypothalamus in Brahman heifers. Nguyen et al., 2017 confirmed the role of zinc finger genes in a co-expression network using ovarian data of the same Brahman heifers. Although these transcriptomic studies confirmed the complexity of puberty, the correlation between expression levels of mRNA and protein is renowned poor (Gygi et al., 1999; de Sousa Abreu et al., 2009; Carvalhais et al., 2015; Payne, 2015; Edfors et al., 2016). As such, proteomic studies are complementary information, required to advance knowledge beyond differential gene expression or co-expression analyses.

Most studies for understanding puberty-related proteins involved in Brahman heifers were performed in peripheral tissues such as the liver and adipose tissues (Nguyen et al., 2018b; 2018c). Also, Tahir and others (Tahir et al., 2019) have published on the ovarian protein abundances in pre versus post-pubertal Brahman heifers. Although a neuropeptidome was performed on the hypothalamus and pituitary gland of Brangus heifers before and after puberty (DeAtley et al., 2018), there is no previous proteome study in the hypothalamus and pituitary gland of pubertal Brahman heifers. Therefore, this present study was aimed at measuring the abundance of proteins in the hypothalamus and pituitary gland in pubertal Brahman heifers using liquid chromatography-electrospray ionisation tandem mass spectrometry (LC-ESI-MS/MS). Discovering protein abundance profiles at the different pubertal stages in the hypothalamus and pituitary gland could be useful for revealing key targets controlling the complex mechanism of puberty and complementing the previous transcriptomic studies.

## Materials and Methods

### Ethics Statement

Animal use in this study was approved under animal ethics number QAAFI/279/12. Animals were housed at the Gatton Campus facilities of the University of Queensland.

### Animal and Tissue Collection

The heifers used in this study and the assessment of puberty have been described by (Fortes et al., 2016; Nguyen et al., 2017; Nguyen et al., 2018a; Lau et al., 2020). In brief, these heifers were of the same age with no difference in body weight or body condition scores. Pubertal status was defined by the presence of the first *corpus luteum* (CL) and progesterone concentration. Postpubertal heifers were euthanized by stunning with a nonpenetratng captive bolt through the parietal bone of the head around day 15 after observing the first CL. The nonpenetrating captive bolt methodology was used for protecting the integrity of the lower brain tissues as previously described (Cánovas et al., 2014). A pre-pubertal heifer was then randomly selected to pair with a postpubertal heifer on slaughter day. Plasma progesterone concentrations were 0.4 ± 0.2 ng/ml and 2.0 ± 0.7 ng/ml in pre and post-pubertal heifers, respectively.

After euthanasia, the hypothalamus tissue (spanning from the preoptic to the arcuate nucleus) and pituitary gland (including anterior and posterior pituitary gland) tissues were quickly harvested, frozen in liquid nitrogen, and stored at −80°C until tissue processing.

### Protein Sample Preparation

Protein extraction and digestion of heifers’ hypothalamus and pituitary tissues were performed as previously described (Tan et al., 2014; Nguyen et al., 2018b; Nguyen et al., 2018c). Briefly, the hypothalamus and pituitary tissues were manually homogenized into smaller fragments using rigid aluminium foil to protect the tissue and a hammer to “pulverize” the sample. This procedure was performed over a dry-ice bed to preserve the sample and randomize the fragments, ensuring that each sample was representative of the entire tissue. The resulting tissue fragments were transferred into Eppendorf^®^ lobind microcentrifuge tubes (Sigma-Aldrich) where lysis solution (7 M urea, 2 M thiourea, 4%SDS, 10 mM DTT and 1 mM PMSF) was added. The fragments in the solution were sonicated at power level 4 for 10 s. Subsequently, the homogenate was vortexed vigorously for 1 h at 30°C. Following that, 25 mM acrylamide was added to the samples and subsequently incubated for 1 h at 30°C. Next, 5 mM DTT was added to samples in order to quench excess acrylamide. Four volumes of methanol: acetone (1:1) were added, and the samples precipitated overnight at −20°C. The precipitates were subsequently dissolved by adding 50 mM ammonium acetate, and the protein concentrations were measured with a Nanodrop (Thermo Scientific). After measuring concentration, about 100 µg of protein were transferred into a 10 kDa Amicon Ultra 0.5 centrifugal filter device (Merck Millipore). This filter device was then inserted into a collection tube and centrifuged at maximum speed for at least 30 min. Protein was diluted in 50 mM ammonium bicarbonate and then again centrifuged at maximum speed for at least 30 min. Trypsin was used as a protease for digestion of protein solution followed by incubation overnight at 37°C. Peptides were desalted by C-18 Zip-tip (adapted from Millipore procedure) and stored at −20°C until analysis.

### Mass Spectrometry and Data Analysis

The LC-ESI-MS/MS was performed using a Prominence nanoLC system (Shimadzu) and TripleToF 5600 mass spectrometer with a Nanospray III interface (SCIEX) as previously described (Xu et al., 2015). Peptides were separated using a 70-min LC gradient. MS-TOF scan was performed. We performed information-dependent acquisition (IDA) of top peptides for one randomly chosen pre-pubertal sample and one randomly chosen post-pubertal sample. Subsequently, sequential window acquisition of all theoretical mass spectra (SWATH) was performed on all samples using the prepared IDA library for each group.

Protein Pilot v5.0.1 (ABSCIEX) was then utilized for peptide identification. The bovine protein database was used for peptide mapping and retrieved from Uniprot (www.uniprot.org; 43,813 entries assigned to *Bos taurus*). For subsequent analyses, identified peptides with more than 99% confidence and a false discovery rate (FDR) of less than 1% were used. Ion libraries were used for SWATH analyses. The abundance of proteins and peptides was computed by PeakView v2.1 software (ABSCIEX). The differential expression analysis between the two grops of pre and post-pubertal heifers as performed using MSstats (v2.6) in R (Choi et al., 2014). For controlling the false discovery rate, the *p*-values were adjusted using the Benjamini and Hochberg’s approach. Proteins with an adjusted *p*-value < 0.01 were assigned as differentially abundant.

### Transcriptomic Data

The hypothalamus and pituitary gland transcriptomic data were obtained from Fortes et al., 2016 and Nguyen et al., 2017. These transcriptomic studies used the same tissues harvested from the same heifers at pre- and post-puberty. The mRNA and protein pairs were identified from sets of expressed genes (Fortes et al., 2016; Nguyen et al., 2017) and set of abundant proteins (current study). The Pearson’s correlation coefficient (*r*) was used to compute the correlations between expression levels of mRNAs and the abundance of proteins in the hypothalamus and pituitary gland from pre- and postpubertal Brahman heifers. The cor() function in R was used in this calculation. The unmatched mRNA-proteins were not included in this analysis.

### Functional Enrichment Analysis of DA Proteins

The gene ontology analysis of differentially abundant (DA) proteins was obtained through STRINGv10 system (http://string-db.org). The DA proteins were classified according to biological process (BP), cellular component (CC), molecular function (MF) and pathways. Further, Cytoscape software (http://cytoscape.org/) was used to retrieve biological terms for the protein interaction networks. In order to determine significant terms and pathways, the terms having a corrected *p*-value (FDR) < 0.05 were considered.

## Results

### Protein Identification, Quantification, and Differential Abundance

**FIGURE 1:**
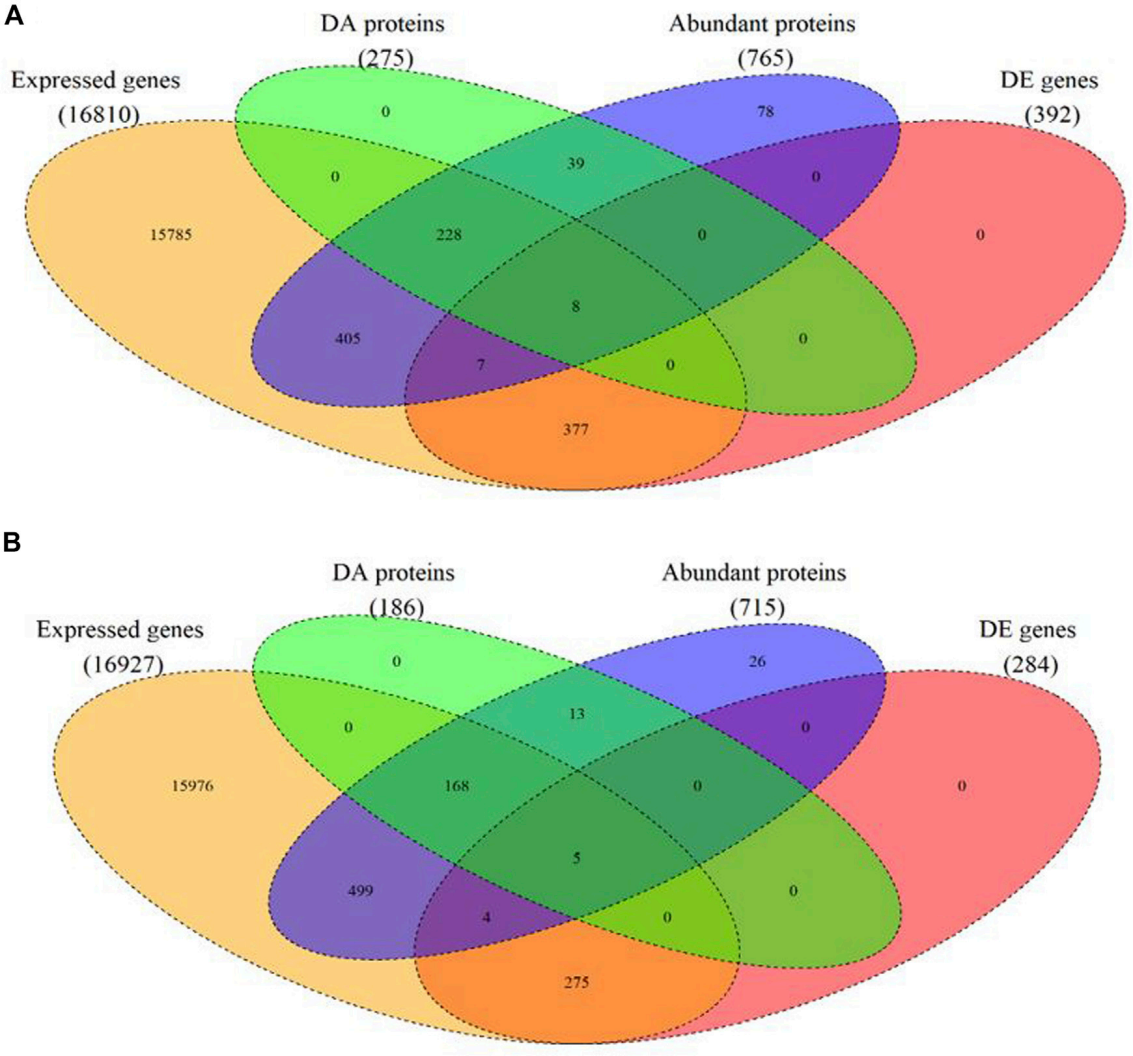
Venn diagram of expressed mRNAs and proteins as well as differentially expressed (DE) mRNA and differentially abundant (DA) protein in each tissue. **(A)** in the hypothalamus tissue, **(B)** in the pituitary gland.

**FIGURE 2:**
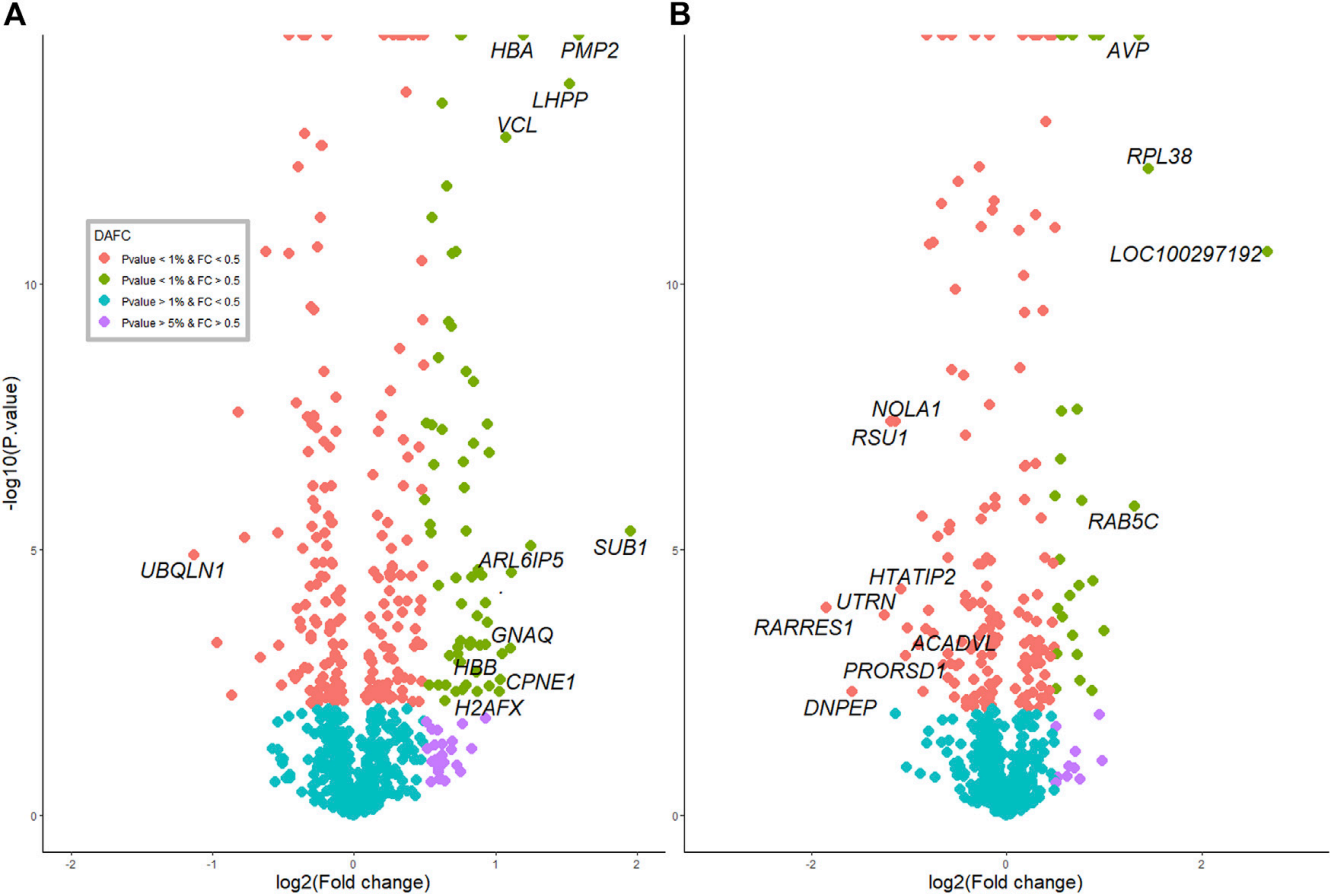
Protein profiles and overlap with RNAseq between pre- and postpubertal heifers in each tissue. **(A)** in the hypothalamus tissue. **(B)** in the pituitary gland.

In total, 765 proteins were identified and quantified in the hypothalamus. Of this total, 275 differentially abundant (DA) proteins were identified (adjusted *p*-value < 0.01) in the hypothalamus libraries, of which 120 were significantly downregulated and 155 were significantly upregulated at post-puberty (adjusted *p*-value < 0.01) (Figures 1A, 2A and Supplementary Table S1).

In the pituitary libraries, a total of 715 proteins were identified and quantified. Of these, 186 were DA proteins, where 96 proteins were significantly decreased, and 90 proteins were significantly increased at post-puberty (adjusted *p*-value < 0.01) (Figures 1B, 2B and Supplementary Table S1).

The extent of DA proteins between pre and post-puberty in each tissue was visualized in volcano plots (Figures 2A,B). Protein IDs, corresponding gene names and fold change (*log*_*2*_FC) information are listed in Supplementary Table S1.

### Derived Correlations Between mRNA Levels and Protein Abundances

The matching pairs of expressed mRNA and proteins in each tissue were used for comparison because of the relatively small number of matched DE mRNA and DA proteins. In the hypothalamus, 641 out of 765 identified proteins corresponded to mRNA transcripts discovered in the hypothalamic transcriptome data (Fortes et al., 2016) (Figure 1A and Supplementary Table S2). The correlation between 641 protein and transcript pairs in the hypothalamus was positive but insignificant, with a correlation coefficient of 0.05 (*p*-value = 0.16). Among these pairs, only eight DE genes were DA proteins, namely *TUBB2B, OMG, SPTAN1, RAP1A, SSBP1, PLCB1, EIF3J* and *H2AFX* (Table 1). Six of these eight DA proteins showed the same direction of regulation as their mRNAs.

**TABLE 1:** List of common differentially expressed genes and abundant proteins in the hypothalamus and pituitary studies.

Tissue	Ensembl ID	Uniprot ID	Gene symbol	FC_mRNA	FC_protein
	ENSBTAG00000004093	Q6B856	*TUBB2B*	−0.203	−0.394
HYP	ENSBTAG00000025213	Q0IIH3	*OMG*	0.199	−0.297
	ENSBTAG00000015327	E1BFB0	*SPTAN1*	−0.131	−0.117
	ENSBTAG00000014710	P62833	*RAP1A*	0.113	0.087
	ENSBTAG00000010931	F1N1S0	*SSBP1*	0.189	0.321
	ENSBTAG00000008338	P10894	*PLCB1*	−0.319	0.779
	ENSBTAG00000000359	G8JKV2	*EIF3J*	0.119	0.845
	ENSBTAG00000038047	Q17QG8	*H2AFX*	0.199	1.026
	ENSBTAG00000005596	F1N2P8	*IGFBP2*	−0.615	−0.452
PIT	ENSBTAG00000007196	V6F957	*TAGLN*	−0.405	−0.698
	ENSBTAG00000011782	P23389	*CHGB*	−0.266	−0.653
	ENSBTAG00000013411	F1MB08	*ENO1*	−0.126	−0.170
	ENSBTAG00000032456	F2Z4I6	*HIST2H2AC*	0.680	−0.338

FC, means fold change; HYP, means Hypothalamus; PIT, means Pituitary.

When compared the proteins in the pituitary gland with its corresponding mRNA data (Nguyen et al., 2017), 648 out of 715 mRNA-proteins pairs were identified (Fortes et al., 2016) (Figure 1B and Supplementary Table S2). The correlation between transcript and protein in pituitary for 648 proteins was minimal (*r* = 0.006; *p*-value = 0.8). Among these pairs, five DA proteins and DE genes were in common. They were: *IGFBP2, TAGLN, CHGB, ENO1* and *HIST2H2AC* (Table 1). Four of these five proteins showed the same direction of regulations as their mRNAs.

### Gene Ontology Enrichment Analysis

In order to evaluate the major biological processes influencing puberty onset in Brahman cattle, GO enrichment was performed to examine the functional characteristics of DA proteins between pre and post-pubertal samples in each tissue (Supplementary Tables S3, S4).

**FIGURE 3:**
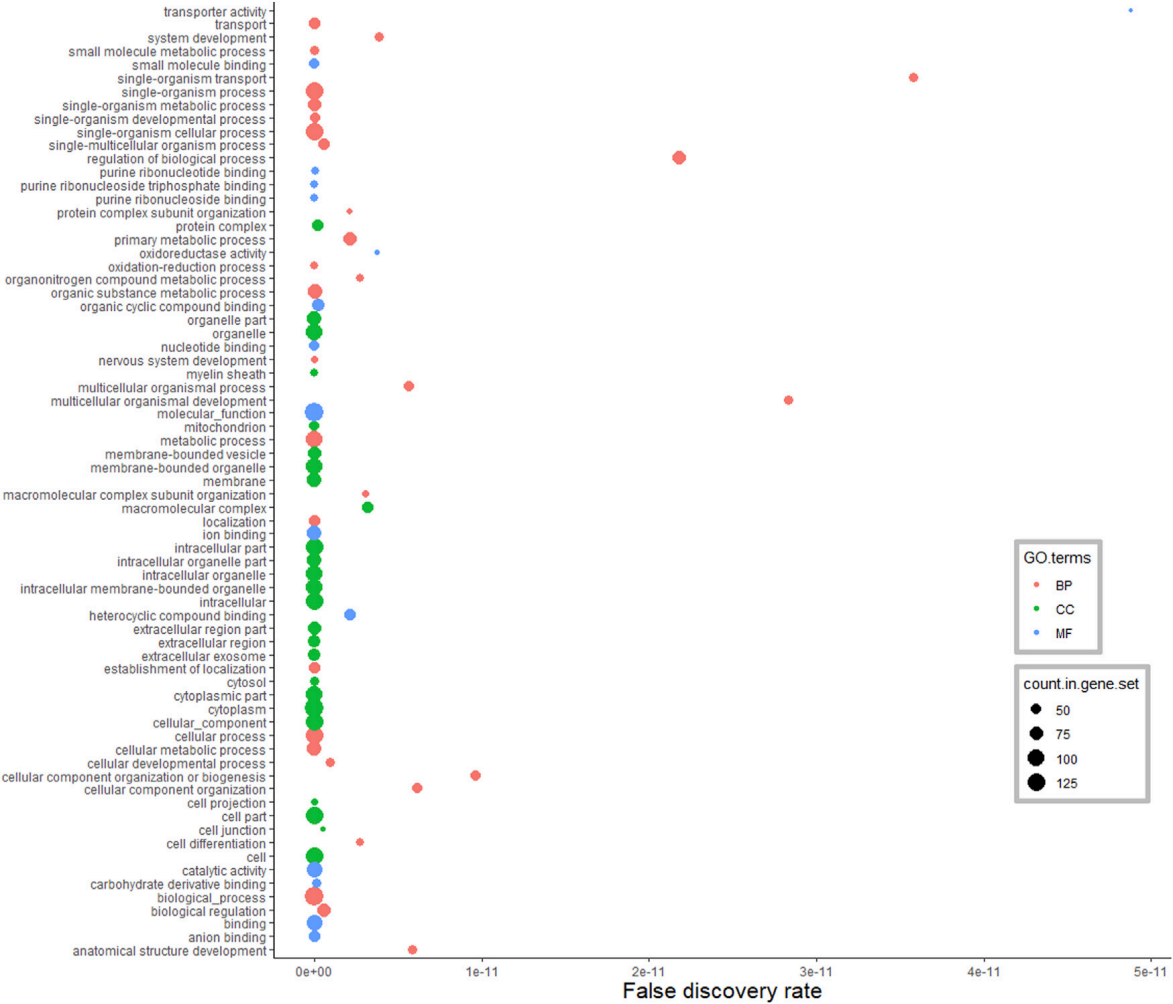
Functional classification of differentially abundant proteins in the hypothalamus between pre-versus postpubertal heifers. Only highly significant GO terms were shown (FDR <1.0E−10).

As shown in Figure 3, DA proteins in the hypothalamus were predominantly enriched for “organic substance metabolic process” (31%), “single-organism metabolic process” (27%), “oxidation-reduction process” (14%) and “nervous system development” (12%), in the BP category of GO terms. As expected, DA proteins were involved in the peptide metabolic process and brain development (including development of neurons, glial cells and axons). These DA proteins were also annotated to the terms “response to oxidative stress,” “glutamine metabolic and catabolic process,” “energy derivation by oxidation of organic compounds,” and “regulation of stress-activated MAPK cascade” (FDR <0.05). MF analysis revealed DA proteins were connected to 89 enriched GO terms, including proteins involved in binding (38%) and catalytic activity (34%). There were 116 enriched GO terms found in the CC category (FDR <0.05), and the majority of DA proteins were located in the cytoplasmic (51%) and intracellular (45%) space.

**FIGURE 4:**
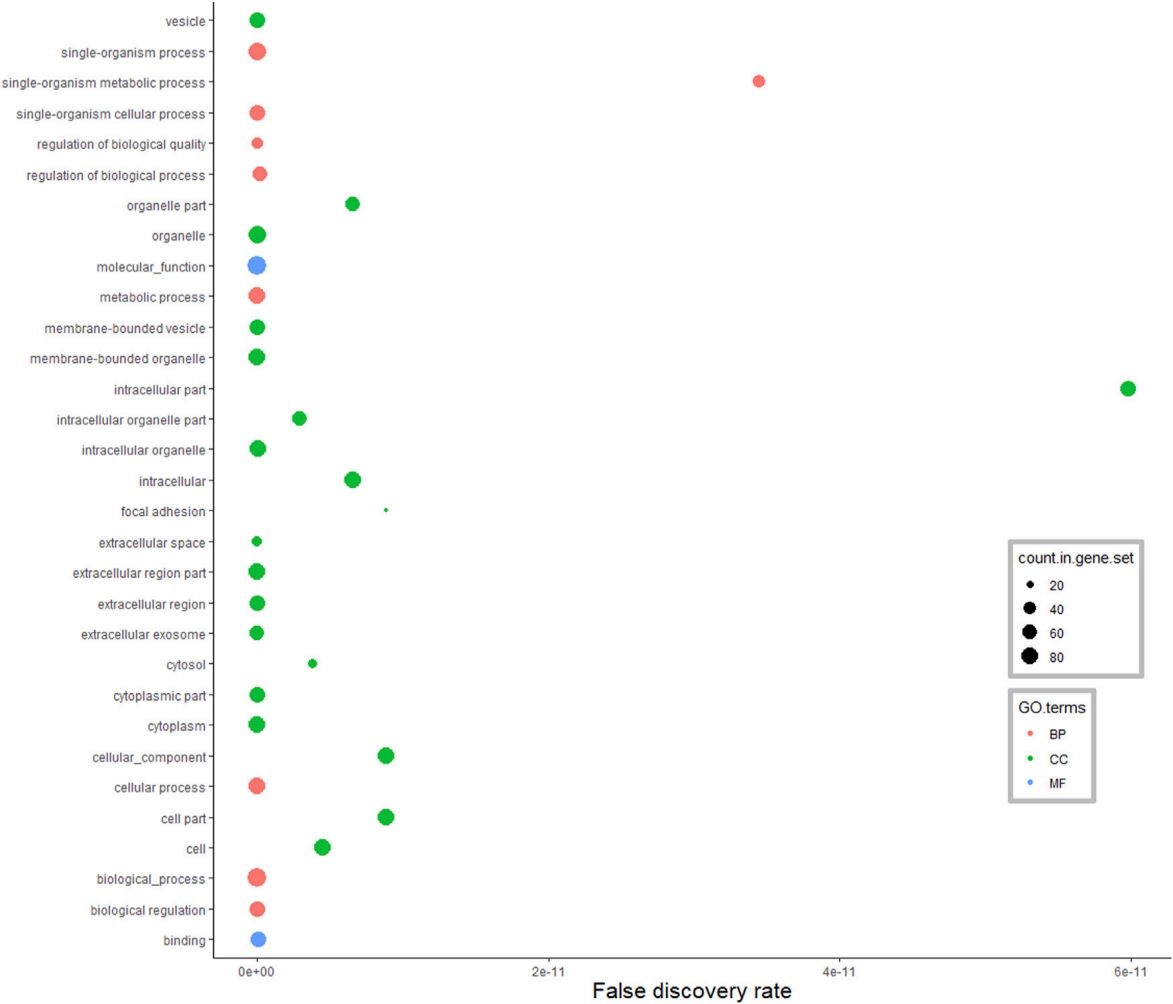
Functional classification of differentially abundant proteins in the pituitary between pre-versus postpubertal heifers. Only highly significant GO terms were shown (FDR <1.0E−10).

According to the GO annotations of DA proteins from the pituitary gland (Figure 4), among 214 enriched BP terms, the majority of DA proteins were classified in the single-organism cellular process (39%), cellular process (38%), metabolic process (35%) and biological regulation (30%). Enriched MF terms were 39 in the analysis of the pituitary gland (FDR <0.05). Hydrolases, oxidoreductases and endopeptidase were the main protein classes identified in this MF anotation. Moreover, the CC terms of pituitary DA proteins were assigned to the organelle, membrane-bounded organelle, cytoplasm, intracellular and extracellular regions.

### KEGG Pathway Analysis

In order to investigate the biological pathways enriched in response to puberty onset in Brahman heifers, a KEGG pathway enrichment analysis was performed.

**FIGURE 5:**
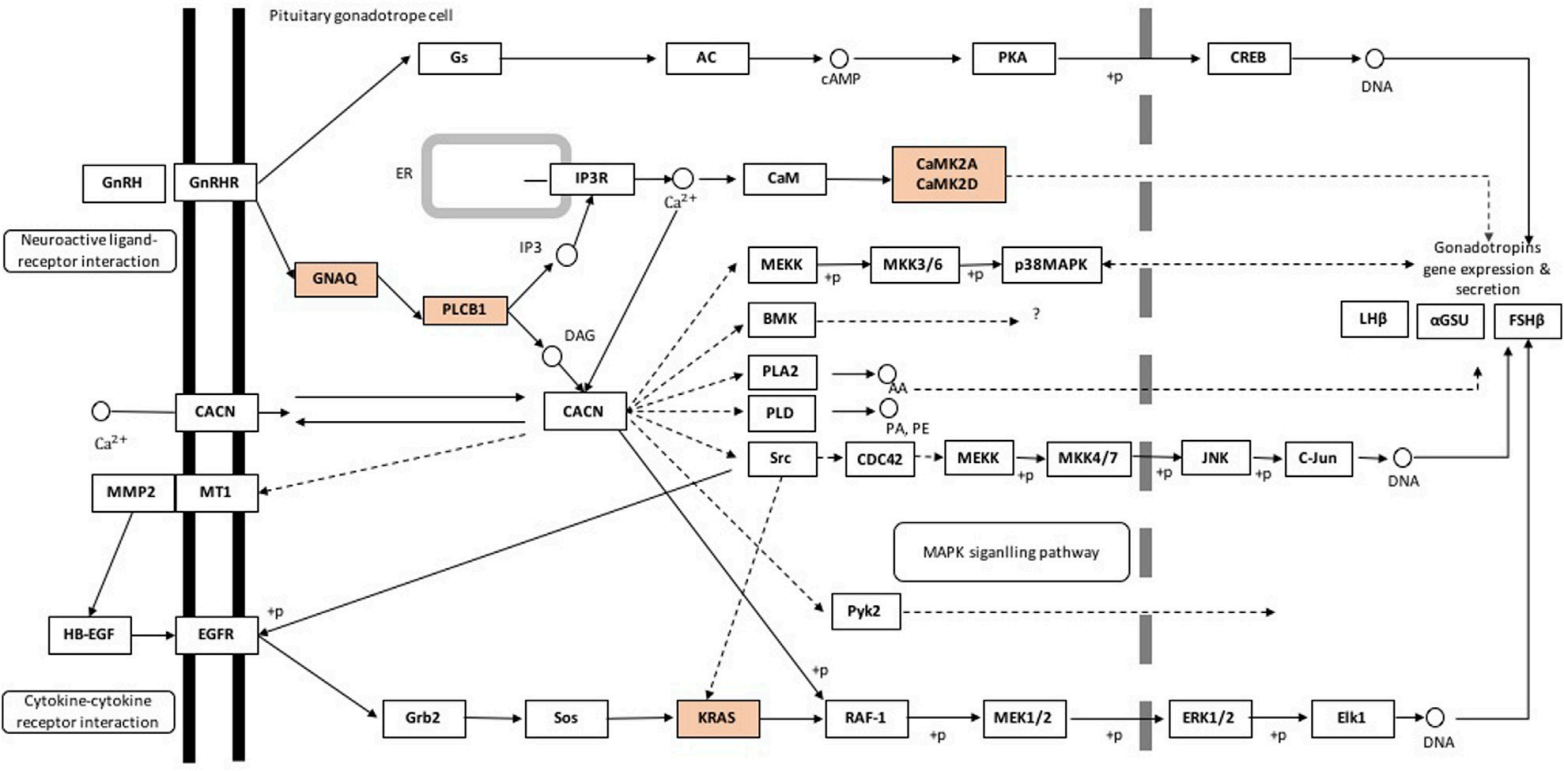
Gonadotropin-releasing hormone signalling pathway containing differentially abundant proteins in the hypothalamus of pre- and postpubertal brahman heifers. Pathway components representing differentially abundant proteins are in orange. Pathway was adapted from the KEGG database (http://www.genome.jp/kegg-bin/show_pathway?bta04912, accessed: 11/03/2018).

In the hypothalamus, comparison of pre- and post-pubertal samples, yielded a total of 275 DA proteins mapped to 67 enriched pathways (Supplementary Table S5). Among enriched pathways, known pathways that are related to puberty were also identified. The enriched pathways included the *estrogen signalling pathway* (FDR = 0.002), *axon guidance* (FDR = 0.006), *glutamatergic synapse* (FDR = 0.0001), *GABAergic synapse* (FDR = 0.005), *GnRH signalling pathway* (FDR = 0.02), *oxytocin signalling pathway* (FDR = 0.001), *calcium signalling* (FDR = 0.01) and *PPAR signalling pathway* (FDR = 0.05). DA proteins involved in the *GnRH signalling pathway* were highlighted in Figure 5. In addition, DA proteins were also involved in other pathways such as *oxidative phosphorylation* (FDR = 6.6 × 10^−12^), *the tricarboxylic acid cycle* (TCA, FDR = 3.58 × 10^−9^), *non-alcoholic fatty liver disease* (NAFLD, FDR = 1.08 × 10^−5^), *glycolysis and gluconeogenesis* (FDR <0.05), *thyroid hormone synthesis* (FDR = 0.05), *metabolism of amino acids* and *carbohydrate metabolism* (FDR <0.05). The DE gene and corresponding DA protein, *PLCB1*, was annotated to four enriched pathways: *GnRH signalling*, *estrogen signalling*, *calcium signalling* and *oxytocin signalling*. In short, pathways related to neuronal-hormonal signalling and metabolism were significant for the DA proteins in the hypothalamus (Table 2).

**TABLE 2:** Enriched pathways related to differentially abundant proteins in heifer puberty in the hypothalamus.

Pathway name	Count	False discovery rate	Proteins involved
GnRH signalling pathway	5	0.02	**GNAQ**, **PLCB1**, **CAMK2A**, **CAMK2D**, KRAS
Calcium signalling pathway	8	0.01	**SLC25A6**, **ATP2B3**, **GNAQ**, **PLCB1**, **PRKCG**, **CAMK2A**, **CAMK2D**, **ATP2B4**
Oxytocin signalling pathway	9	0.001	**EEF2**, **PRKCG**, **CAMK2A**, **GNAQ**, **CAMK2D**, **PLCB1**, GNAI2, GNAO1, KRAS
Axon guidance	7	0.006	GNAI2, RAC1, KRAS, CDK5, DPYSL2, **DPYSL5**, **CFL1**
GABAergic synapse	6	0.004	**GAD2**, **GLUL**, **PRKCG**, GNAO1, GNAI2**,** GNB2
Estrogen signalling pathway	7	0.002	HSPA8, GNAO1, KRAS, GNAI2, HSP90AA1**, PLCB1**, **GNAQ**
PPAR signalling pathway	4	0.05	DBI, ILK, **APOA2**, FABP7

Low and high abundant proteins at post-puberty (adjusted *p*-value < 0.01) appear in normal and bold type, respectively.

**FIGURE 6:**
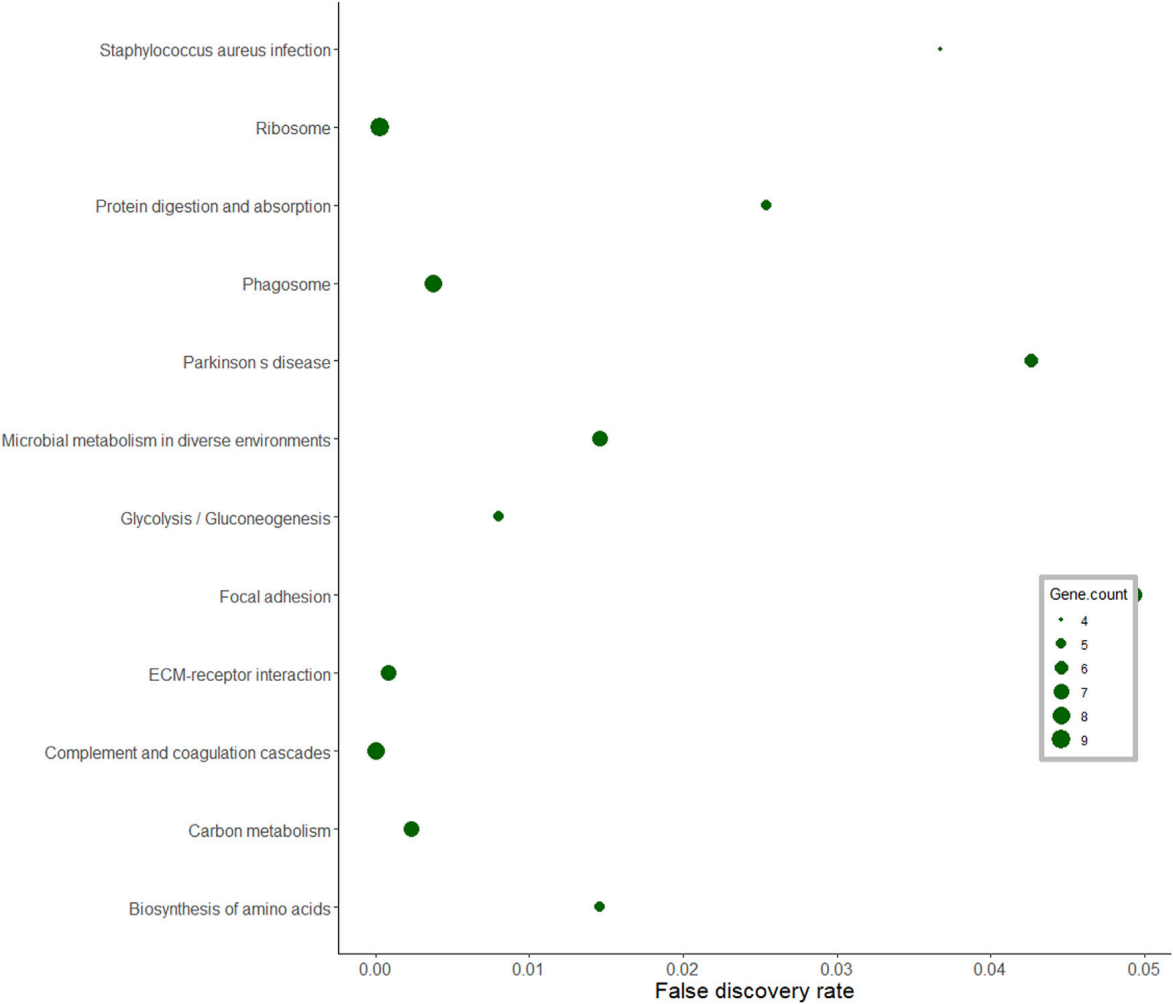
KEGG pathway analysis of differentially abundant proteins in the pituitary gland of pubertal Brahman heifers.

In the pituitary gland, a total of 186 DA proteins were assigned to 12 KEGG pathways (FDR <0.05). Among these enriched pathways, the *ribosome pathway* represented the largest number of DA proteins (FDR = 0.0002). *Complement and coagulation cascades*, *glycolysis and gluconeogenesis*, *biosynthesis of amino acids*, and *focal adhesion* were also overrepresented (Figure 6).

### Protein -Protein Interaction Network Analysis

**FIGURE 7:**
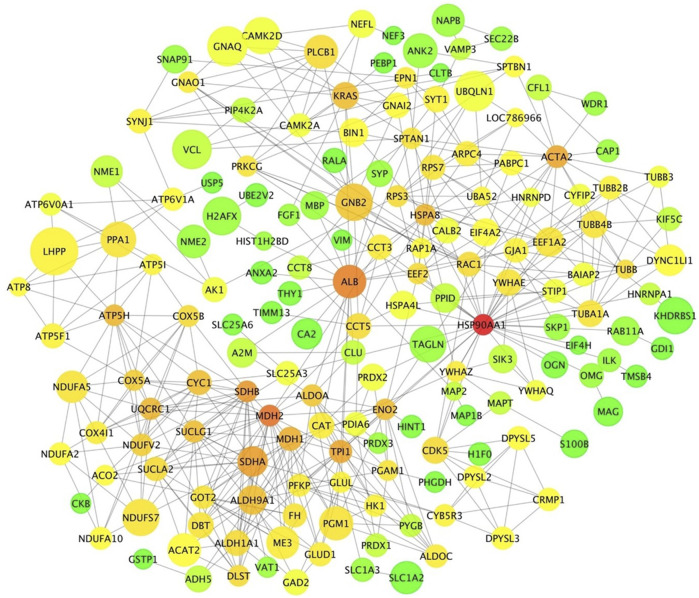
Protein-protein interaction network in the hypothalamic tissue. Each node represents a protein. Edges illustrate the interaction between proteins. Node size represents the level of differential abundance measured for each protein in absolute terms, where bigger nodes represent higher fold changes. Node colour range from green to red represent low to high connectivity in the network for each specific protein.

The relationship among DA proteins was further investigated in a network analysis. The hypothalamus network contained 181 nodes (proteins) with 486 edges (interactions) (Figure 7). Heat shock protein 90 alpha family class A member 1 (*HSP90AA1*) had the highest number of connections in the network (34 edges), followed by malate dehydrogenase 2 (*MDH2*) and albumin (*ALB*) with 23 and 21 connections, respectively. Of note, *HSP90AA1* contributes to the *estrogen signalling pathway*, according to the KEGG pathway analysis (Table 2). In addition, *KRAS* protein, which plays a role in *GnRH signalling*, *axon guidance*, *estrogen signalling* and *oxytocin signalling*, interacted with 13 proteins in the network (Table 2 and Figure 7). PLCB1, a DE gene and DA protein, had nine connections in the network: *GNAQ, GNAI2, PIP4K2A, GNAO1, SYNJ1, GNB2, PRKCG, CAMK2D, CAMK2A* (Table 2 and Figure 7). These genes that were hubs in the network analysis and were associated with enriched pathways of biological relevance for puberty might be essential drivers of puberty in cattle.

**FIGURE 8:**
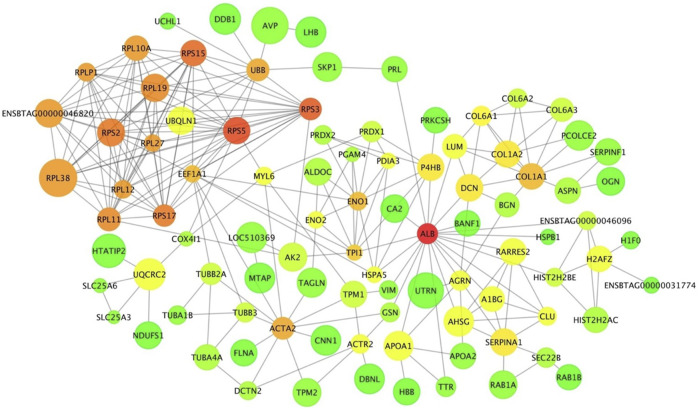
Protein-protein interaction network of differentially abundant proteins between pre- and postpubertal heifers in the pituitary gland. Each protein is a node and protein-protein interactions are illustrated as edges that link nodes. The size of node represents the increase or decrease in protein abundance post-puberty. Node colour represents the connectivity of a protein within the network; it is ranged from green to red for low to high connectivity.

The interaction network from the pituitary DA proteins comprised 113 nodes with 254 edges (Figure 8). Among these nodes, ribosome-related proteins seem to form a dominant cluster in the network. The protein RPS5 had 17 connections within the network. A common DE gene and a DA protein, *ENO1*, was found in the network with 10 connections. This protein is involved in carbon metabolism, biosynthesis of amino acids and glycolysis/gluconeogenesis, as identified by the KEGG pathway analysis of DA proteins in the pituitary between pre- and post-pubertal Brahman heifers.

## Discussions

High-throughput techniques such as transcriptomic and proteomic studies are potent approaches to understanding gene expression and regulation. Mowever, no single approach can stand alone if we are to understand the fundamental biology underlying complex traits. Analyses at multiple levels are needed to interpret complex biological systems. In this study, the correlation between protein abundance and mRNA expression in the hypothalamus and pituitary gland was insignificant. To date, reports on the correlation between mRNA and proteins that use high-throughput approaches are scarce. Yeast, bacteria and human cancer data reports found limited correlations between mRNA and protein levels (Anderson and Seilhamer, 1997; Gygi et al., 1999; Chen et al., 2002; Griffin et al., 2002; Ghaemmaghami et al., 2003; Greenbaum et al., 2003; Washburn et al., 2003; Tian et al., 2004; Nie et al., 2006; Jayapal et al., 2008; de Sousa Abreu et al., 2009; Haider and Pal, 2013; Bai et al., 2015; Carvalhais et al., 2015; Payne, 2015; Edfors et al., 2016). A weak correlation of 0.05 was reported for 17 DE genes and corresponding protein levels analysed in the bovine mammary gland (Dai et al., 2017). The weak correlation between mRNA and protein expression levels may result from methodological constraints and biological factors, such as translational regulation and protein *in vivo* half-lives (Maier et al., 2009; Vogel and Marcotte, 2012; Haider and Pal, 2013). Additional data on factors affecting protein levels and in-depth studies, can help address this conundrum.

Hypothalamus transcriptome and proteome analyses in pre- and post-pubertal Brahman heifers revealed eight DE genes that are also DA proteins. Among these DA proteins, tubulin beta 2B class IIb (*TUBB2B*) is involved in neuronal migration and axonal guidance that are prominent brain remodeling mechanisms during pubertal development (Bond et al., 1984; Romaniello et al., 2015). A study in female rats noted that the neurotrophic effects of estrogen on the central nervous system at the onset of puberty were partly mediated by the increase of tubulin beta class II (Rogers et al., 1993). Further, the significant increase of *TUBB2B* and *TUBB2A* mRNA expression in low progesterone endometrium was reported in heifers (Forde et al., 2012). Our study revealed a decreased abundance of *TUBB2B* post-puberty, suggesting the correlation between TUBB2B and steroid hormones. High expression of *TUBB2B* in pre-pubertal Brahman heifers is proposed to play a part in remodeling the brain and how it responds the feedback of ovarian hormones. Changes to the central nervous system are intrincical to pubertal development.

Another protein among the eight that were DA that were also DE genes in the hypothalamus is spectrin alpha nonerythrocytic 1 (*SPTAN1*). The *SPTAN1* gene is known to play a vital role in brain development and epileptic encephalophathy (Wang et al., 2018). This protein was identified as an upregulated gene in the mammary epithelial cells of pre-pubertal female mice compared to post-pubertal mice (Pal et al., 2017). These results in mice seem contrasting to ours, as *SPTAN1* was downregulated in post-pubertal cattle. Species or tissue differences and the definition for puberty (and therefore timing of sampling) could explain such discrepancies. Nonetheless, SPTAN1 is emerging as a candidate for future research targeting puberty machanisnsm in mammals.

Perhaps the Phospholipase C beta 1 (*PLCB1*) was the DA protein, which is also a DE gene, with more evidence of its role in puberty. *PLCB1* plays a crucial role in reproductive physiology. Genome-wide association studies in pigs reported the contribution of *PLCB1* to growth at puberty onset and in the gonadotropin signalling pathway (Meng et al., 2017). Further, *PLCB1* disruption resulted in infertile mice with pleiotropic reproductive defects (Filis et al., 2013). PLCB1 is a crucial factor modulating GPCR signalling, which controls reproductive physiology in mice (Jiang et al., 1997; Kim et al., 1997; Xie et al., 1999; Bohm et al., 2002; Ballester et al., 2004; Filis et al., 2013). Our results serve as evidence that PLCB1 may have a role in puberty onset in *Bos indicus* cattle: it interacts in the predicted network with other genes (GNAQ, GNAI2, PIP4K2A, GNAO1, SYNJ1, GNB2, PRKCG, CAMK2D, CAMK2A) related to GnRH signalling and other hormonal and neuronal signalling pathways known to be involved in the feedback mechanisms driving the activation of HPO axis. More importantly, PLCB1 was one of the upregulated DA proteins at post-puberty involving in *GnRH signalling*, *estrogen signalling*, *calcium signalling* and *oxytocin signalling*. In hypothalamic neurons, the pulsatile release of GnRH is depended on voltage-gated calcium influx (Krsmanović et al., 1992). Therefore, it is logical to propose that the increase of GnRH release at the hypothalamus of Brahman heifers may be initiated *via* oxytocin signalling, *via* PLCB1.

Pituitary transcriptome and proteome analyses revealed five differentially expressed genes: *IGFBP2, TAGLN, CHGB, ENO1* and *HIST2H2AC,* significantly less abundant post-puberty. Among these, *IGFBP2* was the DA and DE gene with mounting evidence of its roles in puberty. *IGFBP2* is a predominant insulin growth factor binding protein synthesised and secreted in the anterior pituitary during the pre-ovulatory and early luteal phase in beef cattle (Funston et al., 1995; Roberts et al., 2001). In the anterior pituitary, the expression of *IGFBP2* fluctuated with changes in the estrous cycle that were associated with serum progesterone (Funston et al., 1995). In addition, estrogen signalling increased *IGFBP2* expression in the anterior pituitary of ewes, cattle, pigs and rats (Michels et al., 1993; Clapper et al., 1998; Roberts et al., 2001; Rempel and Clapper, 2002). Roberts et al. (2001) reported that IGFBPs levels in the anterior pituitary decreased from pre-ovulatory to early luteal development in beef cattle. Consistent with Robert et al. (2001) observations, the mRNA expression of *IGFBP2* was decreased in the transcriptome study (Nguyen et al., 2017) and the current study. In short, we observed a lower abundancy of *IGFBP2* in the bovine pituitary gland at the luteal phase post-puberty. The release of *IGFBP2* in the pituitary gland was stimulated by gonadotropin-releasing hormone, as demonstrated by Robert et al. (2001). Also, polymorphisms in *IGFBP2* were associated with age at puberty in Brahman cattle (Fortes et al., 2013b).

Chromogranin B (*CHGB*), another DA protein that was also a DE gene from the pituitary studies, belongs to a chromogranin protein family, which has been noted to affect the secretion and storage of FSH and LH at different periods of estrous cycle in sheep (Crawford and McNeilly, 2002). A study of granin-gonadotropin interactions in sheep observed a decrease of *CHGB* mRNA level after the pre-ovulatory LH surge (Crawford and McNeilly, 2002). In agreement with the sheep study, mRNA expression of *CHGB* decreased at post-puberty in the pituitary transcriptome of Brahman heifers (Nguyen at el., 2017). Additionally, the neuropeptide *CHGB* was selected as one of the fertility-related neuropeptides in the pituitary gland neuropeptidome of pre- and post-pubertal Brangus heifers (DeAtley et al., 2018). The neuropeptidome study of Brangus heifers also reported higher levels of *CHGB* neuropeptide products in pre-pubertal pituitary tissue (DeAtley et al., 2018). This study observed a lower abundance of *CHGB* protein after puberty in Brahman heifers. Although measuring neuropeptides is different from measuring protein levels, both studies point to the importance of CHGB in the pituitary glands during pubertal development in cattle.

The DA protein α-enolase (*ENO1*) is one of the three isoforms of glycolytic enzymes responsible for catalysing the conversion of 2-phosphoglycerate to phosphoenolpyruvate (Pancholi, 2001). A study in the ovaries of pre-laying and laying geese suggested that *ENO1* may regulate reproductive function in female geese (Ji et al., 2014; Kang et al., 2014). The overexpression of *ENO1* in granular cells induced the mRNA expression of FSH and LH receptors (Ji et al., 2014). Activation of these receptors is necessary for the hormonal functioning of pituitary glycoprotein hormones (LH and FSH). Pituitary FSH and LH concentrations decreased at puberty in heifers (Desjardins and Hafs, 1968). Our current and transcriptome studies (Fortes et al., 2016; Nguyen et al., 2017) found lower mRNA and protein expression of *ENO1* post-puberty. On the contrary, single-cell RNA profiling found *ENO1* to be down-regulated in a pre-puberty gene network of mouse mammary glands (Pal et al., 2017). It is apparent that *ENO1* impacts female puberty, but its precise function needs to be considered in terms of tissue and species-specific.

The DA protein coded by the DE gene *HIST2H2AC* in the pituitary, a member of the histone 2A family, was mapped to a genetic locus reported linking puberty timing and pubertal height growth in humans (Cousminer et al., 2013). A genetic correlation between age at puberty and high height in Brahman heifers was also reported (Vargas et al., 1998). Differential expression of *HIST2H2AC* at mRNA and proteins level between pre- and post-pubertal heifers proposes it has a role in pubertal development. This candidate gene could be mined for mutations that could be tested for their effects on height and puberty in cattle.

The activation of HPO axis culminates with the pulsatile release of GnRH from the hypothalamus. GnRH triggers the synthesis and secretion of LH and FSH in the pituitary gland, which stimulates the production of gonadal hormones leading to ovulation (Bliss et al., 2010). Both positive and negative feedback at several levels regulate HPO function. One positive feedback on the activation of GnRH secretion is commenced by GnRH receptor (GnRHR) - G-protein α_q_ subunit (Gα_q/11_), which induces increased intracellular calcium (Khadra and Li, 2006). In agreement with this positive feedback, our proteomic study in the hypothalamus revealed the up-regulation of the calcium signalling pathway (FDR = 0.05) and GnRH signalling pathway (FDR = 0.02). G protein subunit alpha q (GNAQ), phospholipase C beta 1 (PLCB1), calcium/calmodulin-dependent protein kinase II alpha (CAMK2A), and calcium/calmodulin-dependent protein kinase II delta (CAMK2D) showed increased abundance at post-puberty in Brahman heifers and were associated with the upregulated pathways. As a result, enrichment analysis of DA proteins from the hypothalamus confirmed the involvement of these proteins on the onset of puberty *via* multiple pathways.

Among enriched GO terms in the pituitary libraries, six of these 11 GO terms, including regulation of multicellular organismal process, extracellular region part, extracellular matrix, proteinaceous extracellular matrix, calcium ion binding and extracellular matrix structural constituent were also found as GO terms involved in the onset of puberty in goat and rats (Gao et al., 2018). These findings and the referenced literature suggest the involvement of these GO terms in the regulation of puberty in goats, rats and Brahman heifers. It seems that many of the mechanisms of female puberty are conserved across mammals.

The interaction network in the hypothalamus revealed three DA proteins as highly connected hubs: HSP90AA1, MDH2 and ALB. The protein HSP90AA1 was involved in estrogen signalling, and it interacted with other proteins of the PPAR and oxytocin pathways (ILK, EEF2 and PRKCG). Recently, a transcriptomic study in pre-versus post-pubertal mammary epithelial cells noted *HSP90AA1* as a DE gene (Pal et al., 2017). Even though there is little evidence of a relationship between MDH2 and female reproduction, MDH2 was characterised as a protein marker for male fertility (Kwon et al., 2015a; Kwon et al., 2015b). A study in Egyptian boys suggested ALB status involved in each puberty stage (Cole et al., 1982). The increase of albumin excretion rate in the urinary tract was significantly associated with puberty in non-diabetic children and adolescents (Bangstad et al., 1993). Bovine serum albumin–estrogen compounds impacted GnRH1 neuronal activity (Temple and Wray, 2005). The inferred network predicted interactions between ALB and proteins involved in estrogen (HSPA8) and oxytocin (EEF2) signalling.

Gao et al. (2018) performed KEGG analysis of long non-coding RNA targets in the hypothalamus of pubertal rats and reported significant enrichment for the ribosome pathway. In this current study, the ribosome pathway was also enriched, and the proteins in this pathway were less abundant post-puberty in the pituitary gland. Further, these ribosomal proteins formed a dominant cluster in the pituitary interaction network. A study in pubertal Brangus heifers observed high expression of the ribosomal protein L39 gene in the pituitary gland (Cánovas et al., 2014). Further, ribosomal DA proteins in the interaction network, such as RPS5, RPS3 and RPLP1, were also listed in the networks constructed from endometrial gene expression of low and high fertility heifers (Killeen et al., 2014). Recently, miR-503-3p was proposed as a new repressor of the initiation of puberty in female mice (Tong et al., 2018). The stable overexpression of miR-503-3p in the GT1-7 cell line can influence ribosome biogenesis pathways and result in down-regulation of puberty-related genes (Tong et al., 2018). The ribosomal protein RPL22 inhibited the expression of Lin28B, a puberty-related gene (Ong et al., 2009; Rao et al., 2012). Another ribosomal protein, namely RPS7, also regulated the PI3K and MAPK signalling pathways that are involved in puberty (Bliss et al., 2010; Acosta-Martínez, 2011; Wang et al., 2013; Nelson et al., 2017; Pal et al., 2017). From the pituitary protein-protein interaction network, there were seven ribosomal proteins that interacted with UBB protein. Disruption in the UBB gene in both male and female mice resulted in infertile animals (Ryu et al., 2008). These collective evidences suggested proteins in the ribosomal pathway are relevant for pubertal development in mammals. Further work could target the DA ribosomal proteins and their targets in the pituitary gland to investigate the molecular mechanisms of puberty in *Bos indicus*.

## Conclusion

In summary, The study confirmed the poor global correlation between mRNAs and proteins, suggesting the need for multiple omics analyses for interpreting complex biological systems. A total of 275 and 186 DA proteins, between pre-pubertal and post-pubertal Brahman heifers in the hypothalamus and pituitary gland were identified, respectively. These DA proteins may regulate puberty onset directly or indirectly. DA proteins in the hypothalamus were mainly associated with metabolic pathways, energy metabolism, and nervous system development. In agreement with previous findings of complex feedback effects on GnRH release, the KEGG pathway analysis indicated that these DA proteins are involved in pathways that convey both inhibitory and excitatory inputs for hypothalamic neurons. In the pituitary, the abundance of proteins involved in protein digestion and absorption, focal adhesion, and ECM-receptor interaction were significantly increased post-puberty. The decreased abundance of ribosomal proteins post-puberty can be interpreted in the context of these genes’ inhibitory input to puberty-related genes. Proteins related to energy production and amino acid biosynthesis may have a crucial role in the neuroendocrine regulation of the pubertal process, meriting further investigation.

## Data Availability

The proteomic datasets for the hypothalamus and pituitary gland have been submitted into Proteome Xchange Consortium *via* PRIDE partner repository with identifier PXD009620 and PXD009619, respectively.
